# Antimicrobial and Anesthetic Niosomal Formulations Based on Amino Acid-Derived Surfactants

**DOI:** 10.3390/molecules29122843

**Published:** 2024-06-14

**Authors:** Martina Romeo, Zakaria Hafidi, Rita Muzzalupo, Ramon Pons, María Teresa García, Elisabetta Mazzotta, Lourdes Pérez

**Affiliations:** 1 Department of Pharmacy, Health and Nutritional Sciences, University of Calabria, Via P. Bucci, 87036 Arcavacata di Rende, Italy; martina.romeo@unical.it (M.R.); rita.muzzalupo@unical.it (R.M.); mazzotta-elisabetta@libero.it (E.M.); 2 Department of Surfactants and Nanobiotechnology, Institute for Advanced Chemistry of Catalonia (IQAC-CSIC), 08034 Barcelona, Spain; zhatnt@cid.csic.es (Z.H.); ramon.pons@iqac.csic.es (R.P.); teresa.garcia@iqac.csic.es (M.T.G.)

**Keywords:** niosome, amino acid-based surfactant, antimicrobial activity, phenylalanine

## Abstract

Background: This work proposes the development of new vesicular systems based on anesthetic compounds (lidocaine (LID) and capsaicin (CA)) and antimicrobial agents (amino acid-based surfactants from phenylalanine), with a focus on physicochemical characterization and the evaluation of antimicrobial and cytotoxic properties. Method: Phenylalanine surfactants were characterized via high-performance liquid chromatography (HPLC) and nuclear magnetic resonance (NMR). Different niosomal systems based on capsaicin, lidocaine, cationic phenylalanine surfactants, and dipalmitoyl phosphatidylcholine (DPPC) were characterized in terms of size, polydispersion index (PI), zeta potential, and encapsulation efficiency using dynamic light scattering (DLS), transmitted light microscopy (TEM), and small-angle X-ray scattering (SAXS). Furthermore, the interaction of the pure compounds used to prepare the niosomal formulations with DPPC monolayers was determined using a Langmuir balance. The antibacterial activity of the vesicular systems and their biocompatibility were evaluated, and molecular docking studies were carried out to obtain information about the mechanism by which these compounds interact with bacteria. Results: The stability and reduced size of the analyzed niosomal formulations demonstrate their potential in pharmaceutical applications. The nanosystems exhibit promising antimicrobial activity, marking a significant advancement in pharmaceutical delivery systems with dual therapeutic properties. The biocompatibility of some formulations underscores their viability. Conclusions: The proposed niosomal formulations could constitute an important advance in the pharmaceutical field, offering delivery systems for combined therapies thanks to the pharmacological properties of the individual components.

## 1. Introduction

Lidocaine is a local anesthetic drug widely used in clinical practice to reduce or eliminate pain, both for surgery and for the management of other forms of acute or chronic pain. However, lidocaine, being a lipophilic compound, has poor permeation through the skin, which constitutes the main obstacle to its application in the field of transdermal administration [1,2]. Nanotechnology has led to the production of nanomaterials as carriers of active substances that amplify their efficacy. Indeed, nanoscale engineering can overcome the limitations associated with traditional drug delivery methods. A good drug delivery system (DDS) acts as a protective shield for the payload, preventing premature decomposition in the biological environment while improving bioavailability, the duration of blood circulation, cellular absorption, and therapeutic activity over longer periods [3,4]. Recently, our group prepared lidosomes: innovative vesicular systems prepared with lidocaine [5]. They were found to be able to self-associate, forming nanosized vesicle structures with good stability. Therefore, lidocaine can be defined as a “surfadrug”, i.e., a pharmacologically active molecule with amphiphilic properties. Chemically, surfadrugs feature one or more flexible hydrophobic aromatic moieties, which may have an ester group or a charged nitrogen atom directly bonded to them, or include a nitrogen atom similar to that in pyridine. It is this flexibility of the aromatic ring that makes these drugs behave similarly to typical surfactants in their associative behavior [6]. It has been observed that these vesicular aggregates improve skin permeation and lidocaine drug deposition on the skin and can act as a capsaicin transporter. Capsaicin (CA) is another analgesic, antioxidant, and antitumoral drug [7]. This compound acts on skin nociceptors involved in pain signals.

Nowadays, infections caused by bacteria resistant to multiple drugs are among the top three global health threats [8]. Adding antimicrobial surfactants to new therapeutic vesicular systems can help reduce the spread of infectious diseases. Cationic surfactants derived from amino acids have emerged as promising, eco-friendly antimicrobials. These compounds possess an amino acid on the polar group and a lipophilic aliphatic chain [9,10]. Their amphiphilic properties, positive charge, and high surface activity enable these cationic agents to disrupt bacterial cell membranes, thereby hindering the development of resistant bacteria. Furthermore, unlike quaternary ammonium compounds (QACs), they can be designed to be biodegradable in the environment, and, consequently, they show lower ecotoxicity.

**Figure 1:**
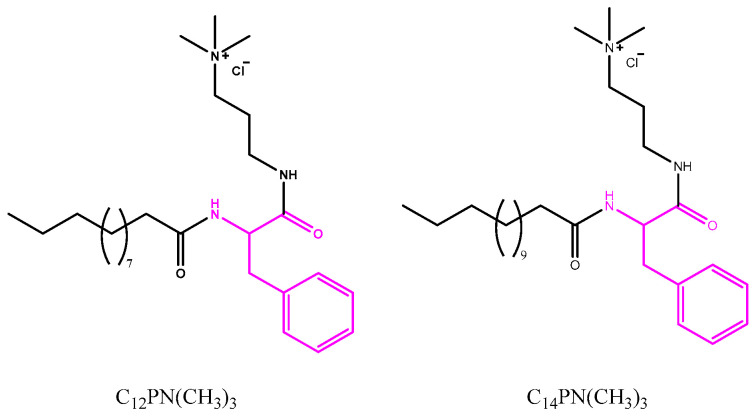
Chemical structure of new antimicrobial cationic surfactants based on phenylalanine.

Recently, our group reported the synthesis of new cationic amino acid-based surfactants based on tryptophan and phenylalanine. The cationic charge of these compounds was situated on a protonated amine group, and, consequently, their cationic character depended on the pH. It was found that these new surfactants formed unilamellar vesicles with DPPC mixtures [10]. Surfactants with a C_12_–C_14_ alkyl chain exhibited good antimicrobial activity; moreover, vesicles containing 60–80% of surfactants maintained their antimicrobial effectivity. Considering these findings, in this work, we prepared similar phenylalanine cationic surfactants, but, in this case, the cationic charge was situated in a trimethylated amine group, meaning that the charge would not depend on the pH (Figure 1). We characterized these new surfactants, and we prepared niosomes containing lidocaine, capsaicin, and the cationic surfactants in order to obtain a colloidal formulation with both anesthetic and antimicrobial properties. The physical characteristics of the new formulations, the vesicle size and size distribution, the zeta potential, the entrapment efficiency, and the stability were determined. The antimicrobial effectivity of both the pure surfactants and their niosomal formulations was determined. Finally, the biocompatibility of these new formulations was studied.

## 2. Results and Discussion

**Figure 2:**
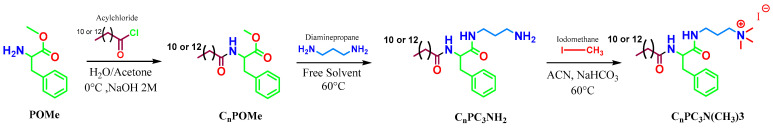
Synthesis procedure of trimethylated phenylalanine-based surfactants, C_n_PN(CH_3_)_3_.

Cationic surfactants based on quaternary ammonium groups, such as CTAB or HTAB, have been widely used as antimicrobial agents in hospitals and in the food industry. Nowadays, the use of these compounds is questioned because they are prepared using petrochemical feedstock, and, in addition, these surfactants exhibit very low biodegradation levels in the environment [11]. In this context, there is a need to develop new antimicrobial surfactants through environmentally friendly approaches to reduce their carbon footprint. Cationic amino acid-based surfactants may be an interesting alternative, given that they show good antimicrobial activity and can be designed to be rapidly biodegradable compounds [12]. From an economic and environmental viewpoint, single-chain surfactants with one amino acid and one polar head are desirable because they can be prepared without using complex procedures that require the use of huge quantities of organic solvents. With these considerations in mind, in this work, we prepared two cationic single-chain amino acid-based surfactants. Their synthesis was carried out in three steps: first, the free amine group of the phenylalanine methyl ester was acylated with the corresponding fatty acid chloride; second, the obtained intermediates were treated with diamine propane to obtain an amide; and, finally, these intermediates were reacted with CH_3_I to obtain the target trimethylated derivative (Figure 2).

The synthesis of these surfactants was carried out considering some of the green chemical principles: the use of renewable resources, a synthetic route consisting in three straightforward steps, reactions which do not require the use of dangerous organic solvents, and the use of non-protected amino acid to avoid extra deprotecting steps. Moreover, the hydrophobic chain was linked to the amine group of the aromatic amino acid through amide bonds to ensure both good biodegradability and great chemical stability. The purity (>96%) was checked by HPLC, ^1^H-NMR, ^13^C-NMR, and HPLC-MS (Supplementary Materials).

### 2.1. Physical–Chemical Characterization of Niosomes

The incorporation of different biologically active compounds can create multifunctional nanocarriers with promising potential applications in biomedicine. Here, we have created for the first time niosomes containing two different anesthetic drugs (lidocaine and capsaicin) and an antimicrobial surfactant. In recent years, amino acid-based surfactants have attracted great interest as they are biocompatible, biodegradable, and environmentally friendly compared to conventional surfactants. Given the different types of amino acids and their different nature, it is possible to design different amphiphilic structures with specific properties [13,14,15]. Vesicles composed of LID, C_14_PN(CH_3_)_3_ or C_12_PN(CH_3_)_3_, and CA have been formulated. Due to the vesicular structure of these aggregates, they can be used and considered by themselves as drug delivery systems. Formulations with different percentages of every component were prepared in our study to modulate the physicochemical and biological properties of these vesicular systems. The vesicles were always prepared using thin-layer evaporation, and their physicochemical properties were evaluated by a Z-sizer instrument in terms of size distribution, polydispersion index, ζ-potential, lidocaine percentage (ELD %), and surfactant encapsulation percentage (EP_n_ %), parameters which may influence their biological activity (Table 1). Additionally, DPPC was also incorporated to study the effect of this phospholipid on the niosome properties.

The L and L/C niosomes showed a medium size (290–350 nm) with similar IP and negative ζ-potential values. These results agree with what was observed recently by our group [5]. Notice that the size of niosomes or vesicles is crucial in transdermal drug delivery systems. Ideally, the size must be below 300 nm to be able to pass through the deepest skin layer. 

First, we will focus on the two niosome formulations containing C_12_PN(CH_3_)_3_. The L/C/P_12_ niosomes had a medium size (280 nm), with a positive ζ-potential due to the presence of the positively charged surfactant. L/D/P_12_ exhibited a lower diameter size, with low positive ζ-potential values; in this case, the C_12_PN(CH_3_)_3_ derivative did not offset the negative character of LID.

For the C_14_PN(CH_3_)_3_ homologue, seven different formulations were prepared: one with LID, two with LID and CA (L/C), another with LID and DPPC (L/D), and three with LID, CA, and DPPC (L/C/D). The physicochemical properties of L/C/P_14_ and L/D/P_14_ were similar to those observed for the analogous C_12_PN(CH_3_)_3_ formulations, the only difference being that the C_14_PN(CH_3_)_3_-based niosomes had higher positive ζ-potential values. The formulations containing all the components (L/C/D/P_14_) displayed comparable sizes (between 260 and 300 nm), with a PI between 0.4 and 0.5 and large positive ζ-potential values (20–23). These parameters indicated a low degree of particle size heterogeneity and sufficiently high particle positive charges for the electrostatic stabilization of the niosomes. All the formulated niosomes were subjected to E% determination to assess the amount of CA and cationic surfactant encapsulated in the aggregates. In general, a high percentage of encapsulation was obtained. It could be observed that the E% of CA was better for the niosomes containing the C_14_PN(CH_3_)_3_ surfactant. Moreover, this percentage increased as the LID% decreased and the DPPC% and C_14_PN(CH_3_)_3_% increased. The formulations containing the four components resulted in better percentages of lidocaine or capsaicin retained in the vesicle structures. It was also observed that the percentage of encapsulation of the P_14_ derivative increased as the proportion of DPPC augmented. 

**Figure 3:**
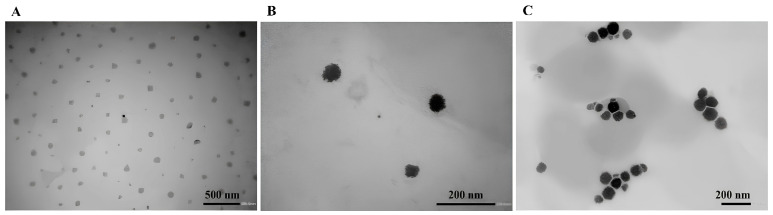
Typical TEM photomicrograph of the lidosomes: (**A**) L/C/P_14_ (7:1:2), (**B**) L/C/D/P_14_ (5:1:2:2), and (**C**) L/C/D/P_14_ (2:1:5:2) formulations. The bar is 500 nm for (**A**) and 200 nm for (**B**,**C**).

**Figure 4:**
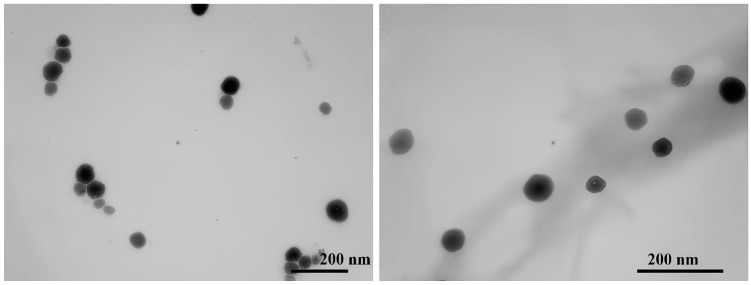
Typical TEM photomicrograph of the lidosomes’ L/C/P_12_ (7:1:2) formulations. The bar is 200 nm.

The morphology of some of these formulations (L/C/P_14_, L/C/P_12,_ L/C/D/P_14_ (5:1:2:2), and L/C/D/P_14_ (2:1:5:2)) was subsequently analyzed by transmission electron microscopy. Figure 3 and Figure 4 show the photomicrographs of these niosomal formulations. The L/C formulation contained spherical vesicles (Figure 3A). The morphological analysis of the lidocaine formulations also showed that this drug formed spherical vesicles that were homogeneous in shape and size [5]. The incorporation of DPPC into the L/C formulation (Figure 3B) did not change the morphology of the aggregates. Finally, it was observed that the formulation containing all the components (L/C/D/P_14_) also showed spherical vesicles homogeneous in size but of an uneven shape. Finally, Figure 4 shows the images obtained for the P_12_ derivate. These vesicles were also spherical in shape, with sharp boundaries. 

### 2.2. Stability Evaluation

The potential use of vesicle formulations is related to their stability at different storage temperatures. The stability of these niosomal formulations was evaluated by determining the average vesicle size, the size distribution, and the entrapment efficiency over one month of storage at room temperature. The results, depicted in Table 2, showed that, in general, these formulations maintained similar dimensions to the starting size. This stability could be ascribed to the medium size of the aggregates and their moderate PI. Moreover, this behavior could be also related to the formation of hydrogen bonds between the components of these niosomes. The cationic surfactants used in this work contained amides that could easily form hydrogen bonds. In this regard, Guo et al. have showed that the formation of hydrogen bonds facilitates the formation of three-dimensional chiral structures [16]. It was also observed that, for almost all the preparations, the percentage of lidocaine, capsaicin, and cationic surfactant entrapped in the aggregates remained stable. However, the E% of the P_12_ derivative decreased significantly after one month: this behavior could be ascribed to the lower hydrophobic character of these compounds compared to P_14_. It was also observed that the initial E% of this surfactant was lower than that obtained with P_14_. It was expected that P_12_, due to its lower alkyl chain, would result in weaker hydrophobic interactions with the other components of the niosome bilayer. For the formulations without DPPC in the lipid film, vesicles polydispersion increased with time, while those containing DPPC maintained the initial values. 

### 2.3. Langmuir Balance Analysis: Surface Pressure/Area Isotherms

Phospholipid monolayers serve as a straightforward model to mimic biological membranes, allowing the study of surfactant–membrane interactions at the air–water interface. The study of the interaction of antimicrobials with lipid model monolayers can help one understand possible mechanisms of antimicrobial activity in which antimicrobial compounds affect the behavior of biological membranes. Given that, in this work, we studied the antimicrobial properties of the formulated niosomes, which entailed the interaction of surfactants–bacterial membranes, we also investigated the interactions of all the niosomal components with simulated biological membranes using a DPPC monolayer at the air–water boundary. The aim of these experiments was to obtain information about surfactants–bacterial membrane interactions. We noticed that, if the surfactant fully detached as the monolayer was compressed, the resulting isotherm would align with that of pure DPPC. Deviations in this behavior indicated that interactions between the surfactant and the DPPC occurred. For this reason, compression π-A isotherms of DPPC/LID, DPPC/CA, DPPC/P_12_, and DPPC/P_14_ mixtures in an 80/20 ratio were obtained with a Langmuir balance. An isotherm of DPPC alone (80/0) was also included as a reference. To reproduce physiological media, the monolayers were spread over PBS at pH = 7.4. The DPPC/π-A isotherm at 25 °C agreed with that reported by Mangiarotti et al. [17]. When the monolayer was compressed, the area decreased, and, then, the gaseous phase (G) changed to a liquid, expanded phase (LE). With further compression, the monolayer state changed to a liquid condensate (LC), and the compression ended with the collapse of the monolayer when π reached 70 mN/m.

**Figure 5:**
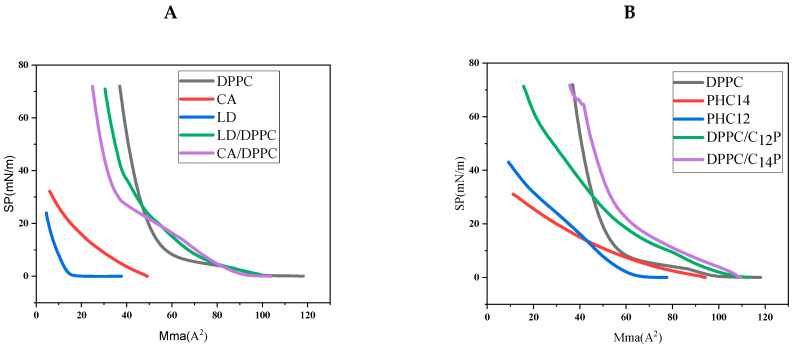
π-A compression isotherms for (**A**) pure LID, CA, and DPPC/CA DPPC/LID mixtures and (**B**) P_12_, P_14_, DPPC/P_12_, and DPPC/P_14_ mixtures.

The π-A isotherms of the niosomal compounds (LID, CA, Figure 5A) alone showed a shape typical of compounds with good water solubility, with spread monolayers almost not compressible. When the monolayers were compressed, the compounds were solubilized in the aqueous phase. For these products, only gas and expanded liquid phases were present in their isotherms, and the collapse occurred at very low surface pressure values.

The isotherms of compounds P_12_ and P_14_ (Figure 5B) exhibited a higher tendency to form expanded monolayers under compression. The presence of alkyl chains in the molecules of these compounds increased their hydrophobic character and decreased their solubility.

Figure 5A,B also contain the compression isotherms corresponding to mixtures of DPPC with every niosomal component. The DPPC/CA and DPPC/LID isotherms (Figure 5A) showed similar phases to that of DPPC. Compared with DPPC alone, the transition from G to LE in these mixtures occurred at the same surface pressure and mean molecular area; however, the transition from LE to LC occurred at higher surface pressures.

At low surface pressure values (<20 mN/m), these mixed monolayers showed an isotherm profile that was shifted toward molecular areas larger than that corresponding to the DPPC monolayer. This could be ascribed to the presence of DPPC/LID or DPPC/CA aggregates in the air–water interface. At higher surface pressures, the isotherm profiles were moved toward smaller molecular areas. For the same surface pressure value, the corresponding area per molecule was smaller. This indicated that both DPPC/LID and DPPC/CA aggregates, under compression, could be forced to solubilize in the bulk solution, resulting in a drop in the number of molecules at the interface.

The presence of P_12_ and P_14_ at the interface resulted in an important DPPC monolayer disorder. Both the DPPC/P_12_ and DPPC/P_14_ isotherms only had G and LE phases. Compared with DPPC, the DPPC/P_12_ isotherm was shifted toward bigger areas, indicating that this surfactant interacted with the phospholipid and was retained at the interface. The DPPC/P_14_ isotherm showed a different behavior. At low surface pressures (π < 30 mN/m), the surfactant also remained at the air–water interface. It is possible that this surfactant formed aggregates with DPPC by means of hydrophobic interactions between the alkyl chain of both compounds. At high surface pressures, the isotherm was shifted to lower molecular areas, indicating that the DPPC/P_14_ aggregates were moved to the buffer aqueous solution. This performance could be attributed to the different hydrophobic group of both surfactants. Due to the longer alkyl chain of P_14_, the hydrophobic interactions of this surfactant with DPPC were greater than those of P_12_, and, then, DPPC could better incorporate in mixed DPPC/P_14_ micelles or vesicles in the bulk solution. The fact that these surfactants form mixed monolayers and aggregates can cause the deformation of the bacterial lipidic membrane wall, with consequent bactericidal activity.

### 2.4. Small-Angle X-ray Scattering (SAXS)

**Figure 6:**
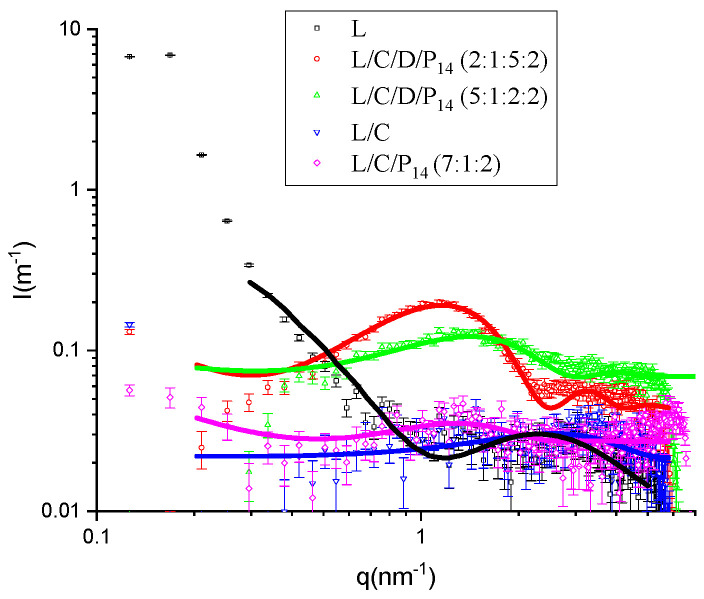
SAXS scattered intensity as a function of dispersion vector modulus q for all the analyzed samples. The lines correspond to the best fit of the bilayer models.

The SAXS spectra were scaled in terms of absolute intensity, and the water spectra were subtracted. The spectra were fitted to bilayer models consisting of two symmetric Gaussians, with the excess electron density representing the polar heads and a central slab with the defect electron density representing the hydrocarbon density [18]. Analyses were conducted on the best experimental formulations that showed good physicochemical properties and promising antimicrobial effects (Figure 6).

Both samples containing DPPC showed the typical band corresponding to phospholipid bilayers (with the maxima around 0.12 nm^−1^ and 0.15 nm^−1^ for L/C/D/P_14_ (2:1:5:2) and L/C/D/P_14_ (5:1:2:2), respectively), but more clearly in the sample with a higher DPPC content. The bilayer corresponding to the sample richer in DPPC showed parameter values closer to those of pure DPPC (in particular, Z_H_ was close to the value obtained for pure DPPC [10]) and better fitting result compared to the one with a lower content (with the Z_H_ value reduced because of the increased proportion of shorter-chain molecule P_14_). This was expected, since we could consider the bilayer to be DPPC with the introduction of some perturbation due to the additives.

**Figure 7:**
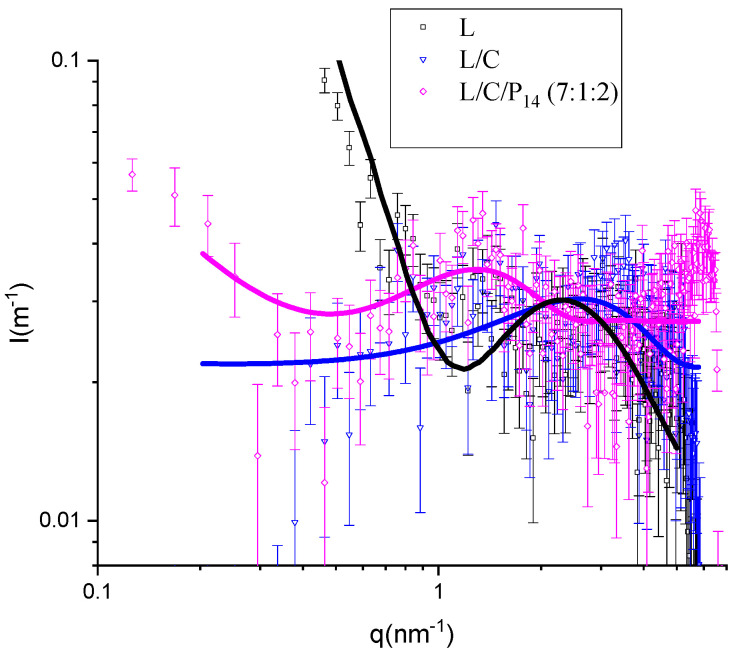
A zoomed-in view of SAXS-scattered intensity as a function of dispersion vector modulus q for L, L/C, and L/C/P_14_ (7:1:2), with the DDPC samples removed.

The other three formulations showed much lower scattered intensities, and the maxima were displaced to larger q values (therefore corresponding to smaller features), as shown in the enlarged view of these three samples (Figure 7). If we were to calculate the volume fraction of the hydrocarbon-like moieties of the different samples, we could observe that the volume fractions of samples L/C/D/P_14_ (2:1:5:2) and L/C/D/P_14_ (5:1:2:2) are 1.6 × 10^−3^ and 1.1 × 10^−3^, respectively, while the lidocaine sample (L) corresponds to 0.84 × 10^−3^, 0.69 × 10^−3^ for L/C/P_14_ (7:1:2), and 0.45 × 10^−3^ for L/C. In this case, SAXS scattering is much lower and with low signal/noise ratios. Nevertheless, the L/C/P_14_ (7:1:2) formulation seems to have some significant information, with a band similar to that of the two DPPC-containing samples. One reason for this behavior can be found in the reduced hydrophobic volume of both lidocaine and capsaicin (0.223 and 0.263 nm^3^, respectively) compared to DDPC (0.866 nm^3^) and P_14_ (0.379 nm^3^) (corresponding to 0.027 nm^3^ for methylene, 0.055 nm^3^ for methyl, and 0.045 nm^3^ for CH=CH, while the hydrophobic volume for lidocaine has been calculated to be that of 1,2,3 trimethyl benzene). This is also congruent with the sizes fitted for the bilayers, the niosomes without DPPC, or P_14_, presenting small bilayer thicknesses (both well-below 1 nm).

**Figure 8:**
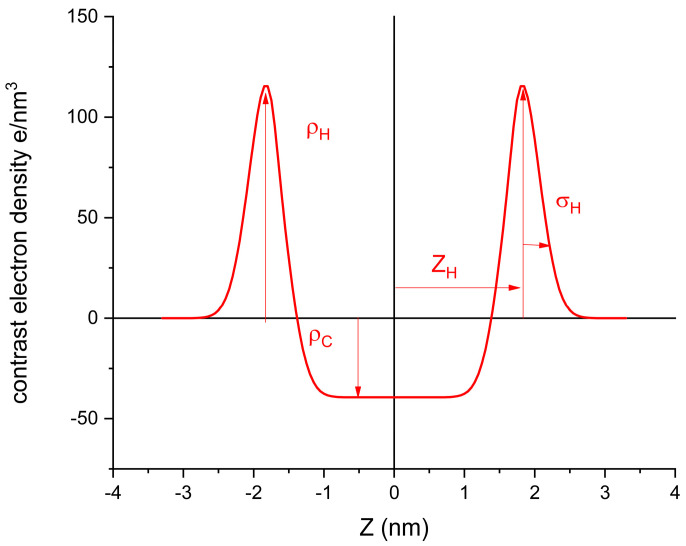
Gaussian-based bilayer profile fitted with the meaning of the different parameters.

Given the large relative error in the measure, due to the low concentrations used, we restricted the fits to this simplified model (no depression in the center of the double layer). To further simplify, we fixed the values of the hydrophobic densities to those corresponding to the volumes shown above (279 e/nm^3^ for the hydrophobic moieties of DPPC and P_14_, 270 e/nm^3^ for capsaicin, and 296 e/nm^3^ for lidocaine). The meaning of the parameters of the model are shown in Figure 8, and the fitted values are shown in Table 3.

The fitting of the lidocaine sample to the bilayers was in contrast with the findings of Shirota et al. [19], who observed, also from SAXs, the formation of small micelles of lidocaine hydrochloride. In their experiment on lidocaine hydrochloride, they found a band present around 8 nm^−1^ in the q range that was not measurable in our experimental setup. However, the size of the micelles, as described by Shirota et al., was far too small. They obtained a hydrocarbon core radius of the micelles of just 0.3 nm, which is not enough to accommodate even a single hydrophobic moiety of lidocaine (0.113 nm^3^ compared to the 0.223 nm^3^ of trimethyl benzene). Given the high level of noise, from the point of view of the SAXS technique, the two samples containing only lidocaine (L) and lidocaine with capsaicin (L/C) were only tentatively fitted to the bilayer model. Other models would provide a similar goodness of fit. What can be stated is that the structures formed were smaller in comparison to the ones in the presence of DPPC. The position of the headgroups, Z_H_, was congruent with those molecules, and lower values were obtained for lidocaine and capsaicin, given that these compounds had shorter hydrophobic moieties than DPPC and P_14_.

**Table 3:** Parameters obtained from fitting the scattering profiles shown in Figure 9. The meaning of the parameters for the Gaussian model are given in Figure 8.

	L	L/C/D/P_14_ (2:1:5:2)	L/C/D/P_14_ (5:1:2:2)	L/C	L/C/P_14_ (7:1:2)
χ^2^	4.5	1.5	1.7	1.5	1.2
σ_H_ (nm)	0.4 ± 0.15	0.46 ± 0.10	0.33 ± 0.10	0.25 ± 0.10	0.62 ± 0.15
ρ_H_ (e/nm^3^)	36 ± 10	111 ± 10	118 ± 10	40 ± 10	65 ± 10
Z_H_ (nm)	0.61 ± 0.15	1.77 ± 0.10	1.44 ± 0.10	0.51 ± 0.10	1.3 ± 0.2

**Figure 9:**
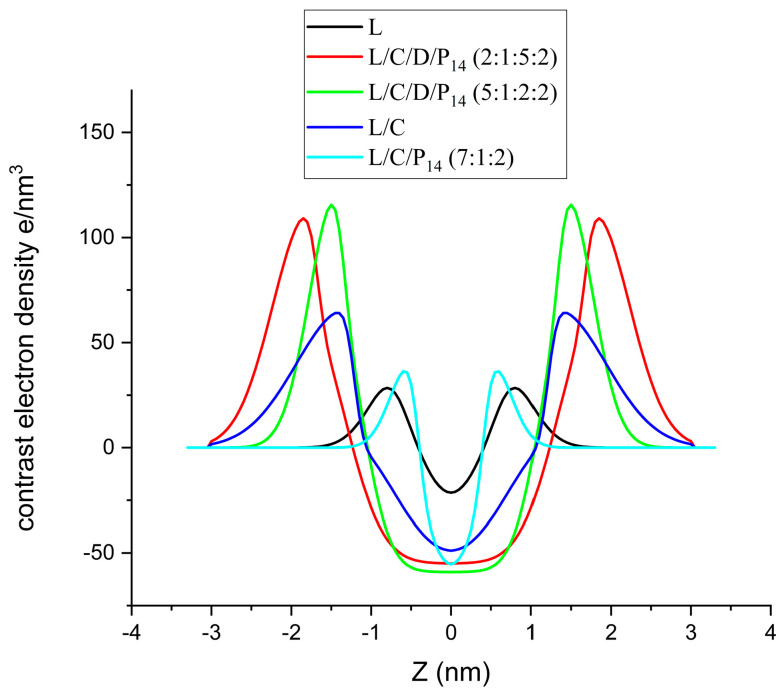
Electronic density profiles obtained from the fitting of the different samples.

According to the literature, LIDHCl forms small micelles in water [19]. At the concentrations used in the experiments here, up to 1.5 mg/mL, 6.3 mM is well-below the reported CMC of ionized species (around 170 mM [20]). Using the neutral form at very low concentrations, water acidity plays a role (more if we consider the spontaneous solubilization of CO_2_); therefore, using the neutral form at low concentrations is equivalent to having a significant amount of LID in the protonated form. In this situation, there are not enough charges in the system to form micelles, but there are enough charges to stabilize the vesicles. The fitting suggests that a plausible model for the observed scattering is that of vesicles with a three-slab electronic density perpendicular to the bilayer and with some polydispersity of the polar head’s width. However, the polydispersity of the hydrophobic slab also produces plausible scattering profiles. The noise does not allow one to decide if using one or other polydispersity parameters is better; however, the electronic density distribution and the overall intensity favor polar head polydispersity.

### 2.5. Antimicrobial Activity

The growing emergence of bacterial resistance requires the development of antimicrobial systems with new modes of action that hamper the growth of resistant pathogens. Because of this, in recent years, much effort has been focused on the development of innovative, safe, and efficient antimicrobial systems. In this work, we developed new amino acid-based surfactants that were incorporated in lidocaine-based niosomes to obtain a new anesthetic system with antibacterial activity. The antibacterial activity was tested against some Gram-positive (*Bacillus subtilis*, *Staphylococcus epidermidis*, *Staphylococcus aureus*, *Listeria monocytogenes*, *Enterococcus faecalis*) and Gram-negative representative bacterial strains (*Escherichia coli*, *Acinobacter baumannii*, and *Klebsiella aerogenes*). The obtained MIC values (concentration required to totally inhibit bacterial growth) as well as the MBC values (concentration of compounds required to kill bacteria) were determined for the pure surfactants and their niosomal formulations.

Table 4 contains the MIC values corresponding to the pure compounds. These two surfactants show antibacterial activity against a wide range of bacteria: the P_14_ derivative is effective against all the microorganisms tested, and P_12_ is active in seven of the eight bacteria assayed. It has been reported that the antimicrobial activity of cationic amphiphiles is related to electrostatic and hydrophobic interactions with bacterial membranes and depends on the hydrophobic character and cationic charge of the surfactant. Firstly, the cationic surfactants interact with the negatively charged compounds of the bacterial membranes; subsequently, the alkyl chain interacts with the lipid bilayers of the membranes and changes their structure, ensuring transport across the cell membranes. As expected, the alkyl chain’s length affects the antimicrobial activity; in our case, the best effectivity was obtained for the P_14_ derivative. The good activity of these compounds can be related to their chemical structure, which is designed to enhance interaction with the lipid bilayers. On the one hand, it has been reported that the presence of aromatic groups can improve the interaction of surfactants with the lipid bilayer of bacterial membranes [21]. Indeed, benzyl ammonium chloride, a widely used antiseptic agent, contains an aromatic group in the polar head. On the other hand, regarding the hydrophobic group, generally, cationic amphiphiles with medium alkyl chains, usually with 12/14 methylene groups, show the best antimicrobial activity. Indeed, these two surfactants showed the best activity against the Gram-positive bacteria. This behavior agrees with their mode of action, as cationic surfactants’ activity mainly involves membrane disruption, and Gram-negative bacteria have a lower permeability of their outer membranes [22]. It should be noted that the development of antimicrobial agents with this mode of action may help reduce the growth of resistant bacteria, given that this mechanism hampers the apparition of resistance.

Table 4 also contains the MBC values of these phenylalanine-based surfactants and the concentration at which >99.9% of bacteria are killed. The obtained MBC/MIC ratio ranged from 1 to 2, indicating that these compounds not only impeded the growth of these microorganisms but also killed them.

Comparing the antimicrobial activity of CnPN(CH_3_)_3_ with that of pH-sensitive surfactants with a similar chemical structure [10], it is observed that all these cationic amphiphiles exhibit a common broad spectrum of antibacterial activity. In this regard, the advantage of these phenylalanine derivatives is that the cationic charge does not depend on the pH, so they are expected to maintain their activity in all pH media. Furthermore, the antimicrobial efficacy of these new cationic surfactants is comparable to that of Benzalkonium chloride (BAC) (Table 4) and QACs with an equivalent hydrophobic group [22].

Table 5 shows the MIC values obtained for some representative niosomal formulations. L/C-based niosomes did not exhibit any activity against the bacterial strains tested. The formulations based on the P_12_ derivative did not exhibit a large difference, and the MIC values for the two formulations were similar. It can be observed that the antimicrobial efficacy of this surfactant was reduced when it was incorporated into niosomal formulations. This behavior could be ascribed to both the size and low ζ-potential value of these niosomes. It is well known that the cationic charge of surfactants is one of the main factors affecting their antimicrobial potency; in fact, increasing the cationic charge density of surfactants is the most common approach to improving the antimicrobial activity of cationic amphiphiles [23]. In this regard, for cationic vesicles based on amino acid surfactants, it has been observed that antimicrobial activity drastically diminishes as positive ζ-potential values decrease [24]. Given its chemical structure, it would be expected for the monocatenary P_12_ derivative to form micelles; however, its niosomes, contained in our study formulations, have large sizes. It has been reported that large aggregates interact worst with biological membranes [25].

Interestingly, the C_14_PN(CH_3_)_3_ encapsulated in these niosomes retained its antimicrobial activity. We noted that the pure surfactant was more active than the P_12_ derivative; moreover, the P_14_-based niosomes had large positive ζ-potential values. It was observed that the niosomal formulations based on this surfactant exhibited very good activity against all the Gram-positive bacteria tested and moderate activity against the Gram-negative ones. These results suggest that niosomes also interact with bacterial membranes, which is very interesting for reducing the growth of resistant bacteria. The niosomes containing L/C/P_14_ and L/D/P_14_ showed similar effectivities, whereas those containing the four components exhibited better activity against the Gram-positive bacteria, probably due to the better cationic charge density on their surface. It was also observed that these niosomes maintained the bactericidal activity of the cationic surfactant.

Liposomes containing antibiotics are widely reported. It has been observed that these liposome systems can improve the stability and antimicrobial activity of these antibiotic compounds [26]. In the literature, works regarding the preparation of liposomes with antimicrobial activity using amino acid-based surfactants and phospholipids can be found [10,27]. Our group has also reported the properties of antimicrobial cationic vesicles based on arginine surfactants [24]. The results of this study indicate that these niosomal formulations mark a significant advancement in the pharmaceutical field due to their dual pharmacological function: one resulting from the antimicrobial activity of the encapsulated cationic surfactant and the other related to the innate anesthetic properties of the lidocaine and capsaicin carriers. 

### 2.6. Biocompatibility of the Formulations: Ex Vivo Hemolytic Activity

**Figure 10:**
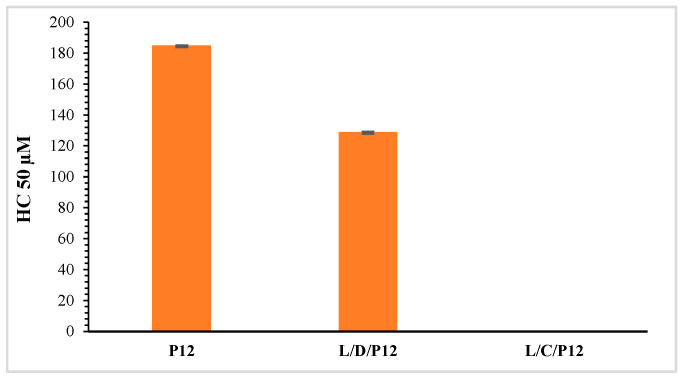
HC_50_ of formulations based on P_12_ (mean ± S.E. of three replicates).

**Figure 11:**
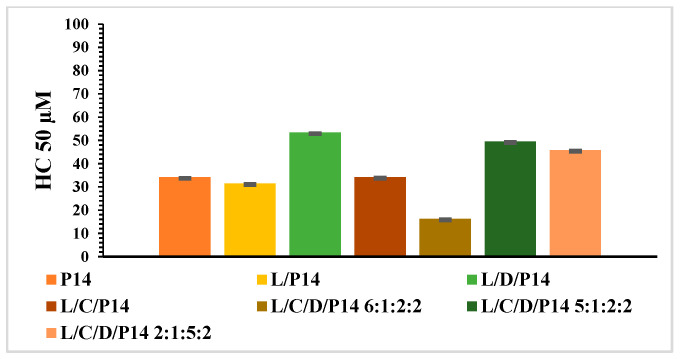
HC_50_ of formulations based on P_14_ (mean ± S.E. of three replicates).

To evaluate the biocompatibility of surfactants and vesicular systems, one of the most frequently used methods is the determination of hemolytic activity. Figure 10 and Figure 11 show the HC_50_ values obtained for the pure surfactants and their formulations. The hemolytic activity of the P_12_ surfactant was very low compared to that obtained for the P_14_ derivative. 

Nowadays, it is still not satisfactorily addressed how the hydrophobicity of surfactants affects their toxicity. In general, when the hydrophobicity of surfactants increases, their hemolytic character also increases due to their stronger membrane permeabilization [28]. However, in some cases, this trend is not followed; for example, Fogt et al. found that monocatenary cationic lipids exhibited a higher toxicity than their double-tailed counterparts [29,30], while Pinnaduwage et al. [31] reported that cetyltrimethylammonium bromide (CTAB) was more cytotoxic and less efficient than the lipid dioctadecyltrimethyl ammonium DOTMA.

The lidocaine vesicles (L and L/C) did not show hemolytic activity in the concentration ranges tested. Non-ionic surfactant-based vesicles (niosomes) are regarded as non-toxic aggregates. The literature highlights the use of blood-compatible amphiphilic surfactants in niosome studies. For instance, niosomes formulated with various non-ionic surfactants, such as Brij 72, Span 20, and Tween 60, have demonstrated minimal hemolytic activity, measuring less than 5% [32]. Also, Ullah et al. prepared non-hemolytic niosomes with a non-ionic surfactant, atocopherol [33].

The niosomes containing the phenylalanine surfactants exhibited hemolytic activity. When niosomes interact with blood, the cationic surface charge seems to be one of the most important influencing factors. The erythrocyte’s membrane contains negatively charged compounds such as glycocalyx sialic acid. Due to their positive charge, cationic niosomes interact with RBCs, altering their shape and causing lysis. Due to this, near-neutral-charge niosomes show better biocompatibility [34]. Given the higher hemocompatibility of the P_12_ derivative, the niosomes containing this cationic surfactant also showed higher HC_50_ values. The L/D/P_12_ (6:2:2) formulation was more hemolytic than the previous formulation, reaching an HC_50_ equal to 129 Μm (Figure 10).

The hemolytic activity of the cationic liposomes based on the P_14_ phenylalanine derivative is shown in Figure 11. The L/P_14_ (9:1) and L/C/P_14_ (7:1:2) formulations showed HC_50_ values comparable to that of the pure cationic surfactant (around 30 µM). The addition of DPPC led to an increase in biocompatibility, reaching HC_50_ values between 46 and 54 μM. The only exception was observed with the L/C/D/P_14_ (6:1:2:1) formulation, which showed an even lower HC_50_ concentration than the pure surfactant solution.

The hemolytic activity of cationic surfactants has been widely reported; however, there are limited data available on cationic niosome surfactants. Hemolysis can occur either due to a disruption of the molecular structure of the red blood cell (RBC) membrane or an increase in the membrane’s permeability to external solutes. This process begins with cationic surfactants being absorbed onto the surface of erythrocytes and their subsequent distribution between the aqueous phase and the membrane. The presence of cationic charges on the niosomes can lead to a significant interaction with the RBC membrane. Additionally, the characteristics of the niosome aggregates can influence hemolytic activity. Vesicle aggregates of different sizes lead to significantly different hemolytic activities [35]. Smaller niosomes tend to be more toxic than larger ones because they more readily interact with RBCs, causing extensive hemolysis. This principle can explain the fact that the hemolytic capacity of the pure P14 surfactant solution was usually higher than that of its corresponding niosomes.

The structural parameters affecting the cytotoxicity of surfactants are similar to those affecting antimicrobial activity. Because of this, cationic surfactants with high antimicrobial activity usually show great hemolytic activity. In this regard, it is very important to develop antimicrobial systems with selectivity against bacterial membranes. The therapeutic index (TI = HC_50_/MIC ratio) indicates the bacterial selectivity. Table 6 shows the TI for the pure surfactants and their niosomal formulations. TI values higher than 1 indicate that the compound or niosome formulation possesses antibacterial activity against the microorganism at concentrations lower than those at which it shows hemolytic activity. It can be observed that the pure surfactant solution shows a TI higher than 1 for all the Gram-positive bacteria tested. This means that this surfactant shows selectivity against Gram-positive bacteria. A better selectivity for the niosomal formulations is obtained for those containing DPPC; they also show selectivity against almost-Gram-positive bacteria. However, neither the pure surfactants nor their niosomal formulations exhibit selectivity against the Gram-negative microorganisms. More studies are necessary to optimize the selectivity of these systems against these problematic bacteria.

### 2.7. Molecular Docking Results

In response to the escalating drug resistance of bacterial strains, various strategies have been explored to hinder the virulence factors of *Staphylococcus aureus* [36]. A key virulence factor of *S. aureus* is the golden carotenoid pigment staphyloxanthin (STX), which serves a crucial role as an antioxidant. The presence of STX has been demonstrated to confer protection against oxidative stress to *Staphylococcus aureus*, as it is biosynthetically generated by the enzyme dehydrosqualene synthase (CrtM) [37,38]. Consequently, targeting the biosynthesis of STX emerges as a promising avenue for therapeutic intervention. Structurally, CrtM is similar to the squalene synthase enzyme (SQS), a human enzyme which participates in cholesterol synthesis [39]. Quinuclidines (or quinuclidinols) are the most potent classes of SQS inhibitors [40], and they are expected to interact with SQS as cationic (ammonium) species [41].

**Figure 12:**
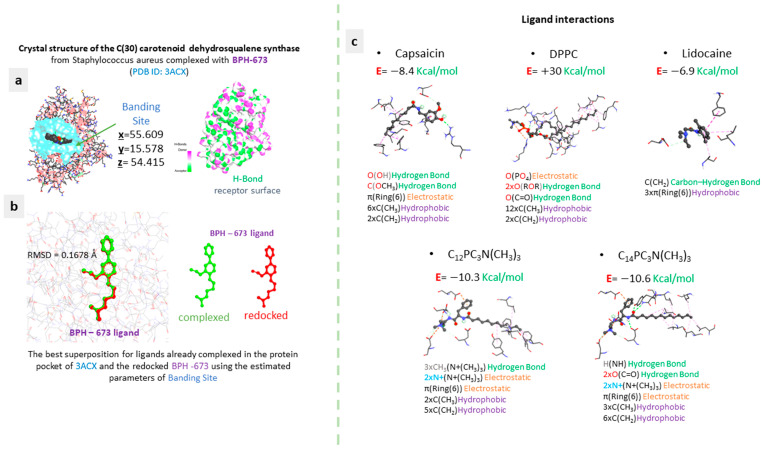
Molecular docking studies detailing the different compositions of the niosome formulations: (**a**) crystal structure of the C(30) carotenoid dehydrosqualene synthase from *S. aureus* (PDB: 3ACX); (**b**) validation of the docking protocol with confirmation by redocking the co-crystallized ligand BPH-673 into the dehydrosqualene synthase active site with the root mean square deviation (RMSD = 0.1678) ≥ 2; and (**c**) illustrations demonstrating the various interaction types observed in the contact modes between the different compositions of niosome formulations and the protein pocket of the receptor 3ACX.

Examining the structural attributes of compounds via molecular docking is pivotal in discerning their affinity for dehydrosqualene synthase (CrtM). This approach sheds light on the mechanism by which these compounds interact with CrtM at a molecular level, offering insights into their efficacy as antibacterial agents. Successful inhibitors of CrtM disrupt the synthesis of vital components within bacterial cell membranes, resulting in either bactericidal or bacteriostatic outcomes. The prospect of specifically targeting bacterial enzymes such as CrtM, while avoiding interference with human counterparts, holds the potential to yield robust antibacterial effects with minimal adverse reactions [42]. The results, depicted in Figure 12, reveal that both surfactants demonstrate docking scores within a specific range, spanning from −10.3 kcal/mol to −10.6 kcal/mol. Additionally, capsaicin (−8.4 kcal/mol) and lidocaine (−6.9 kcal/mol) exhibit affinity with the ligands, suggesting robust interactions. Conversely, a weaker affinity is observed with DPPC, as evidenced by a positive interaction energy. This indicates a thermodynamic disadvantage for the DPPC_CrtM receptor complex. The three types of interactions (electrostatic, hydrophobic, and hydrogen bonding) are consistently observed across all interaction modes. In the case of the two surfactant molecules, their inclusion in the formulation enhances electrostatic interactions due to the presence of the quaternary ammonium group, while the linear hydrophobic chain increases the number of hydrophobic interactions, particularly with methyl groups engaging different receptor residues. Furthermore, DPPC exhibits multiple hydrophobic interactions, with its CH2 groups contacting amino acid residues. However, the positive docking energy mentioned earlier emphasizes the unfavorable nature of these interactions from a thermodynamic point of view. Conversely, lidocaine’s mode of interaction involves only two types of interactions, lacking electrostatic interaction despite featuring a phenyl group, which typically participates in such interactions in the case of capsaicin’s mode of interaction. The outcomes from molecular docking underscore the significance of each ingredient’s presence in formulating niosomes. The inclusion of hydrophobic and cationic surfactants enriches the number of electrostatic and hydrophobic interactions. Consequently, the antibacterial nature of these formulations enables them to confront various enzymes involved in post-bacterial wall destruction, including CrtM; these molecules exhibit favorable thermodynamic affinity and integration-mode affinity with this enzyme. This, in turn, facilitates the inhibition of this enzyme. In essence, the correlation between antibacterial efficacy and molecular docking against CrtM necessitates a thorough comprehension of the interactions between the compounds and the enzyme, alongside their effects on bacterial viability and growth. Incorporating this computational approach in experimental investigations is pivotal in elucidating and refining the development of potent antibacterial agents.

## 3. Materials and Methods

Lidocaine, capsaicin, and dipalmitoyl phosphatidylcholine were purchased from Sigma-Aldrich (Milan, Italy, purity 99%). The organic solvents were supplied by Sigma-Aldrich (Milan, Italy). Muller-Hinton (MH) broth and agar were obtained from Difco Laboratories (Franklin Lakes, NJ, USA).

### 3.1. Synthesis of the Phenylalanine-Based Surfactants

The phenylalanine-based surfactants used in this work were prepared following the synthetic procedure showed in Figure 2. C_n_PC_3_NH_2_ was synthesized using the process outlined in our previous work [10]. In brief, the free amine group of the phenylalanine methyl ester was acylated with dodecyl or tetradecyl acid chloride; then, the C_n_POMe compounds were dissolved in diaminopropane at 60 °C to obtain the corresponding C_n_PC_3_NH_2_. Then, 1 g of the C_n_PC_3_NH_2_ was reacted with methyl iodide (CH_3_I) in a DMF medium to yield the target compound C_n_PN(CH_3_)_3_. The reaction mixture was kept at 60 °C, with the progress being monitored using HPLC. After completing the reaction, excess CH_3_I was removed using a rotary evaporator, and the mixture was purified via preparative HPLC. The identification of the target surfactants was accomplished through HPLC, MS, and NMR analyses. Details of this analysis can be found in the Supplementary Materials.

### 3.2. HPLC Analysis

The reaction development as well as the purity of the prepared surfactants were evaluated by HPLC (Merck-Hitachi D-2500, Merck, Rahway, NJ, USA, UV–vis detector L-4250 at 215 nm, Lichrospher 100 CN column). All the analyses were carried out using a gradient elution program: from 25% to 95% of the organic phase in 24 min. The aqueous phase consisted of 0.1% trifluoro acetic acid in H_2_O (*v*/*v*), while the organic phase contained 0.085% (*v*/*v*) of TFA in H_2_O/CH_3_CN (1:4).

### 3.3. NMR Experiments

Sample solutions were prepared in deuterated methanol (20 mg/0.8 mL) using 5 mm NMR tubes. The ^1^H NMR and ^13^C NMR analyses were recorded on a Varian 400 MHz spectrometer, Varian, Palo Alto, CA, USA. The data were processed with the MestReNova (Mestrelab Research 14.1) software.

### 3.4. Mass Spectroscopy

The surfactant were dissolved in methanol to obtain a concentration of 1 × 10^−4^ to 1 × 10^−6^ M. High-resolution mass spectra (HRMS) were acquired via the Acquity UPLC System and an LCT PremierTM XE Benchtop orthogonal acceleration time-of-flight (Waters Corporation, Milford, MA, USA) equipped with an electrospray ionization source. The obtained data were processed and displayed using the MestReNova software (Mestrelab Research 14.1).

### 3.5. Niosomes’ Preparation

The niosomes were prepared using the film hydration method. An adequate mg of the different ingredients was dissolved in 3 mL of ethanol, and the solvent was evaporated to obtain a thin dry film. The lipid film was then hydrated with 10 mL of distilled water at 60 °C for 30 min. The non-encapsulated ingredients were removed by dialysis using membra-cel dialysis tubings (Visking MWCO 12,000–14,000 pore diameter, SERVA Electrophoresis GmbH, Heidelberg, Germany) for 4 h in distilled water [43]. After purification, the niosomes were stored at room temperature. Details on the vesicle compositions are reported in Table 7.

### 3.6. Physical–Chemical Characterization of Vesicles

The diameter, size distribution, and ζ-potential of the niosomes were measured using a Zetasizer ZS (Malvern Instruments Ltd., Malvern, UK) at 25 ± 0.1 °C. Following purification by dialysis, all the formulations were analyzed; the measurements were performed in triplicate and presented as mean values with standard deviations. The morphology of the niosomes was examined via transmission electron microscopy (TEM) using a TEM ZEISS EM 10 from Oberkochen, Germany.

### 3.7. Langmuir Balance Analysis

The monolayer isotherms were evaluated using Langmuir balance. The surfactant solutions were dissolved in hexane/methanol (9:1) at a concentration of 1 mg/mL and distributed over the aqueous subphase with a Hamilton microsyringe. All the mixtures were in an 80/20 M ratio (DPPC: surfactant). Pure DPPC was used at 80%, thus keeping the concentration of DPPC equal to the mixtures with the surfactants. All the experiments were carried out using a 20 mM buffer as the subphase. After 15 min of evaporation, the π-A isotherms were recorded with a symmetrical barrier compression rate of 20 mm/min. The molecular area was determined as the total area divided by the total number of molecules deposited on the surface.

### 3.8. Small-Angle X-ray Scattering (SAXS)

The SAXS measurements were carried out using S3-MICRO (Hecus X-ray system GMBH Graz, Graz, Austria), following the procedure described in reference [10]. Briefly, all the samples—niosomal and background solutions—were introduced in a flow-through glass capillary 1 mm diameter with a 10 μm wall thickness. SAXS scattering curves as a function of the scattering vector modulus were obtained:

(1) $$q=\frac{4\pi }{\lambda }\sin\left(\frac{\theta }{2}\right)$$

where *θ* is the scattering angle. The *q* values with this setup ranged from 0.1 nm^−1^ to 6.0 nm^−1^. A standard silver behenate sample was used to calibrate the system’s scattering vector. Because of the use of a detector-focused small beam (300 × 400 μm full width at half maximum), the scattering curves were mainly smeared by the detector width and detector depth. This smearing mainly produced a widening of the peaks without a noticeable effect on the peak position in the small-angle regime. The scattering curves were background-subtracted considering the concentration of the solvent and solutes in the samples. The instrumentally smeared experimental SAXS curves were fitted to numerically smeared models for beam size, detector width, and detector depth effects. A least-squares routine based on the Levenberg–Marquardt scheme was used. A model of the scattering by the liposomes was used, based on the description of the bilayer electronic density profile, using a Gaussian for each polar head and an error function to describe the hydrophobic contribution [18]. The errors were determined using the boot-strapping technique.

### 3.9. Determination of the E% of LID, CA, and Cationic Surfactants in Vesicular Systems

The encapsulation percentage of each component was quantified by HPLC; 1 mL of purified niosomes and 1 mL of non-purified niosomes were diluted separately in 100 mL of methanol. The analysis was conducted at 280 nm for CA and 210 nm for LID and the amino acid-based surfactants. The E% of every ingredient was calculated according to the following equation:

E% = C_R_/C_I_ × 100 (2)

where C_R_ is the concentration measured after the niosome’s purification, and C_I_ is the concentration used for the preparation of the vesicles. All the experiments were performed in triplicate and expressed as the mean ± standard deviation (SD).

### 3.10. Stability Evaluation

To determine the stability of the vesicles, the formulations were stored at room temperature (25 ± 2 °C) and evaluated at predetermined time intervals (0 and 30 days) in terms of visual inspection, particle size, PI, E%, and ζ-potential.

### 3.11. Biocompatibility of the Formulation: Ex Vivo Hemolytic Activity

The determination of hemolytic activity was carried out on fresh rabbit blood. We followed an adaptation of the method described by Pape et al. (1987) [44]. The red blood cells were washed three times in a phosphate-buffered solution (PBS pH 7.4). The cells were then suspended at a cell density of 8 × 10^9^ cells per mL. A series of different volumes of the niosomal solution (3760 μg/mL), ranging from 10 to 130 μL, were placed in Eppendorf; 25 μL erythrocyte suspension and phosphate-buffered saline were added to Eppendorf, up to a total volume of 1 mL. The samples were incubated at room temperature while shaking for 10 min. Following Eppendorf, they were centrifuged for 5 min at 10.000 rpm. The percentage of hemolytic activity was determined by comparing the absorbance at (575 nm) of the supernatant of the samples with that of the control hemolyzed with distilled water. Each analysis was performed in triplicate.

The therapeutic index (*TI*) in this context was calculated as the ratio of the concentration at which 50% of the cells were affected (*HC*_50_) to the minimum inhibitory concentration (*MIC*) for each surfactant formulation against both microorganisms and eukaryotic cells. The equation for calculating the therapeutic index (*TI*) was the following:

(3) $$TI=\frac{{HC}_{50}}{MIC}$$

This index provided a quantitative measure of the selectivity of the surfactant formulations against microorganisms relative to eukaryotic cells. A higher *TI* suggested greater selectivity and lower potential toxicity to eukaryotic cells.

### 3.12. In Vitro Antimicrobial Activity

Antimicrobial tests were conducted using bacteria stored in our laboratory. The microorganisms were *Bacillus subtilis* ATCC 6633 (BS), *Staphylococcus epidermis* ATCC 12228 (SE), *Staphylococcus aureus* ATCC 29213 (SA), *Listeria monocytogenes* ATCC 15313 (LM), *Enterococcus faecalis* ATCC 29212 (EF), *Escherichia coli* ATCC 25922 (EC), *Acinetobacter baumanii* ATCC 19606 (AB), and *Klebsiella aerogenes* ATCC 13048 (KA). The antimicrobial activity was determined in vitro to obtain the MIC value, defined as the lowest concentration of antimicrobial agents which inhibits the development of visible growth after 24 h of incubation at 37 °C [45]. The MIC of our niosomes was determined using a broth microdilution assay [46]. A serial dilution of niosomes in Mueller–Hinton broth (MHB) was dispensed, and 200 μL of this was added in the corresponding wells of a 96-well polypropylene microliter plate. Initially, 10 μL of each bacterial strain’s initial culture was added to obtain an inoculum of approximately 5 × 10^−5^ colony-forming units (CFU) per m. The MHB without surfactant was used as a control for bacterial growth. The turbidity in the inoculated wells indicated microorganism growth. To confirm the MIC observed, 20 μL of 0.015% *w*/*v* resazurin was added to each well and incubated for 2 h at 37 °C. Using this indicator, the MIC was shown by the color change from blue to pink. Finally, to determine the minimum bactericidal concentration, the MBC (antimicrobial concentration corresponding to at least a 3-log reduction in viable cells) was obtained as follows: a 10 μL aliquot from the MIC well and the two concentrations above it were plated on MH agar and incubated for 24 h at 37 °C. The MBC was defined as the lowest concentration at which no colonies were detected on MH agar plates.

### 3.13. Molecular Docking

The molecular docking investigations were conducted employing AutodockVina [47,48] to elucidate the binding mode and mechanism of the systems [49]. The 3D crystal structure of the C (30) carotenoid dehydrosqualene synthase in a complex with BPH-673 (PDB ID: 3ACX) served as the target enzyme [50]. The target crystal structure (3ACX) underwent pretreatment to eliminate water molecules, ligands, and other non-proteinaceous entities. H-atoms were added to ensure the proper ionization and tautomeric states of the amino acid residues. AutoGrid was employed to prepare the geometry of the binding site, utilizing a grid box of dimensions (x, y, z) = (16, 38, 16) enclosing the original ligand (BPH-673), with a box spacing of 0.37 Å. Default parameters were applied using Autodock Tools 1.5.4 [51]. The optimal interactions of the protein–ligand complexes were scrutinized using PYMOL and Discovery Studio Client v 17.2.0. Additionally, the reliability of the docking procedure was validated by redocking the co-crystallized ligand BPH-673 into the active site of dehydrosqualene synthase, yielding a root mean square deviation (RMSD) of 0.1678 Å. Figure 2 illustrates the superior alignment of the two ligands upon redocking, which had been initially complexed within the protein pocket of 3ACX. The ligands, C_12_PC_3_N(CH_3_)_3,_ C_14_PC_3_N(CH_3_)_3_, capsaicin, lidocaine, and DPPC were depicted using the Chemdraw 12.0 software. Subsequently, to identify the most stable conformation, the geometry of these ligands was optimized employing the Molecular Force Field (MMFF94) as implemented in the same software.

### 3.14. Statistical Analysis of Data

The data were expressed as the means ± standard deviation.

## 4. Conclusions

The development of antimicrobial niosomes using lidocaine together with phenylalanine derivatives as the primary constituents represents a significant advance in pharmaceutical formulations. Capsaicin was used as a model drug. The niosomal systems formulated in various ratios with P_12_ and P_14_ phenylalanine derivatives and subsequently enriched with DPPC showed sizes between 180 and 300 nm, with a uniform spherical structure. The SAXS analysis showed that the DPPC-containing samples exhibited the typical band corresponding to phospholipid bilayers, with only minor effects due to the additives. The L/C/P_14_ sample (7:1:2) behaved similarly to the two DPPC-containing samples. The other two samples showed much lower scattering intensities, the profiles of which were compatible with reasonable bilayer electronic density profiles. The surface pressure/area isotherms revealed that the presence of P_12_ and P_14_ at the interface significantly disrupted the DPPC monolayer. The phenylalanine derivatives exhibited good antimicrobial activity against Gram-positive bacteria and moderate activity against Gram-negative ones. The designed niosomes, particularly those containing the C_14_ phenylalanine homologue, retained good bactericidal activity against the Gram-positive bacteria associated with the cationic surfactants. The niosomes containing phenylalanine surfactants showed varying degrees of hemolytic activity, with the remarkable fact that some of the vesicles containing the C_14_ derivative and DPPC showed lower hemolytic activity and superior selectivity against almost all Gram-positive bacteria. These results suggest an interaction between niosomes and bacterial membranes, which is promising for combating resistant strains. This study highlights the potential of these niosomal formulations as versatile delivery systems with a dual functionality. Their properties show promising characteristics for antimicrobial applications in pharmaceuticals by harnessing the antimicrobial properties of the encapsulated cationic surfactant, along with the intrinsic anesthetic and analgesic effects of lidocaine and capsaicin.

## Figures and Tables

**Table 1:** Physicochemical characterization of the different vesicular systems in terms of hydrodynamic diameter (SIZE), polydispersion index (PI), ζ-potential (PZ), percentage encapsulation of lidocaine (E% LID), percentage encapsulation of capsaicin (E% CA), and percentage encapsulation of cationic surfactants (E% P_n_) at 25 °C.

* Formulations	SIZE nm	PI	PZ	E% LID	E% P_n_	E% CA
**L**	348 ± 93	0.381	−16.8 ± 4.5	47.6 ± 1.9	-	-
**L/C (9:1)**	294 ± 63	0.393	−9.6 ± 1.3	28.7 ± 5.8	-	56.1 ± 20
**L/D (8:2)**	675 ± 31	0.602	−5.6± 1.5	42.0 ± 2.6	-	-
**L/C/P_12_ (7:1:2)**	279 ± 77	0.446	11.6 ± 0.7	29.8 ± 2.3	67.2 ± 0.6	40.2 ± 3.5
**L/D/P_12_ (6:2:2)**	194 ± 15	0.301	0.406 ± 3.8	27.2 ± 1.4	51.8 ± 0.8	-
**L/P_14_ (9:1)**	289 ± 46	0.284	15.5 ± 0.9	26.4 ± 3.6	68.5 ± 2.4	-
**L/C/P_14_ (7:1:2)**	309 ± 104	0.349	15.7 ± 0.4	31.3 ± 0.3	84.2 ± 1.3	64.3 ± 1.5
**L/D/P_14_ (6:2:2)**	177 ± 8	0.611	15.2 ± 1.2	23.7 ± 2.7	86.9 ± 2.4	-
**L/C/D/P_14_(6:1:2:1)**	302 ± 60	0.564	21 ± 2.5	33.3 ± 0.8	79.7 ± 2.1	70.0 ± 10
**L/C/D/P_14_ (5:1:2:2)**	259 ± 23	0.394	19.6 ± 10	42.8 ± 3.8	81.5 ± 2.5	74.7 ± 1.2
**L/C/D/P_14_ (2:1:5:2)**	260 ± 4	0.583	22.6 ± 1.3	40.5 ± 1.3	85.1 ± 0.1	65.6 ± 0.7

* L (lidocaine), C (capsaicin), D (DPPC), P_12_ (C_12_P(CH_3_)_3_, and P_14_(C_14_P(CH_3_)_3_).

**Table 2:** Stability evaluation of LID formulations stored at 4 °C, expressed in diameter, P.I., ζ-potential, and E% at 25 °C. The data are reported as the mean of three independent experiments.

Formulations	Time	Size	IP	PZ	E% LID	E% P_n_	E% CA
**L/C**	0	294	0.393	−9.6	28.7	-	56.1
	30	1761	0.508	−6.6	36.8	-	31.3
**L/C/P_12_ (7:1:2)**	0	279	0.446	11.6	29.1	67.2	40.2
	30	183	0.505	6.2	33.5	18.7	74.3
**L/C/P_14_ (7:1:2)**	0	309	0.349	15.7	31.3	84.2	64.3
	30	275	0.447	20.6	-	-	-
**L/D/P_14_ (6:2:2)**	0	176	0.611	15.2	23.7	86.9	-
	30	271	0.430	14.0	-	-	-
**L/C/D/P_14_ (6:1:2:1)**	0	302	0.564	21.0	33.3	79.7	70.1
	30	122	0.393	25.1	35.5	82.5	67.1
**L/C/D/P_14_ (5:1:2:2)**	0	258	0.394	19.6	42.8	81.5	74.7
	30	162	0.444	19.0	44.2	87.9	75.5
**L/C/D/P_14_ (2:1:5:2)**	0	260	0.583	22.6	40.5	85.1	65.6
	30	102	0.520	22.2	39.6	84.9	66.7

**Table 4:** MIC/MBC values of pure phenylalanine-based surfactants.

	C_12_PN(CH_3_)_3_ MIC (MBC) (µM)	C_14_PN(CH_3_)_3_ MIC (MBC) (µM)	BAC (µM)
**Gram-positive**			
**BS**	62 (62)	16 (16)	16
**SE**	32 (64)	8 (64)	16
**SA**	32 (128)	8 (16)	16
**LM**	125 (250)	16 (32)	62
**EF**	64 (64)	16 (16)	8
**Gram-negative**			
**EC**	125 (500)	125 (125)	62
**AB**	250 (250)	32 (125)	62
**KA**	>500 (>500)	250 (250)	62

**Table 5:** MIC/MBC values (µM) of formulations based on C_n_PN(CH_3_)_3_.

	L/D/P_12_ (6:2:2)	L/C/P_12_ (7:1:2)	L/D/P_14_ (7:2:1)	L/C/P_14_ (7:1:2)	L/C/D/P_14_ (5:1:2:2)
**Gram-positive**					
**BS**	161 (161)	62 (62)	41 (41)	53 (106)	12 (25)
**SE**	161 (161)	124 (124)	20 (20)	27 (27)	12 (12)
**SA**	80 (80)	62 (124)	20 (20)	54 (54)	25 (50)
**LM**	322 (>322)	249 (>249)	82 (82)	27 (27)	50 (50)
**EF**	80 (80)	124 (249)	41 (41)	54 (54)	25 (25)
**Gram** **-** **negative**					
**EC**	322 (322)	249 (249)	>164 (>164)	54 (54)	100 (100)
**AB**	322 (322)	249 (249)	82 (82)	54 (54)	50 (50)
**KA**	>322 (>322)	249 (249)	>164 (>164)	106 (106)	400 (400)

**Table 6:** Therapeutic index of niosomal formulations.

Microorganism	Therapeutic Index (TI)					
	P_12_	P_14_	L/D/P_12_ (6:2:2)	L/D/P_14_ (7:2:1)	L/C/P_14_ (7:1:2)	L/C/D/P_14_ (5:1:2:2)
**BS**	2.9	2.1	0.8	1.3	0.6	4.1
**SE**	5.8	4.3	0.8	2.7	1.3	4.1
**SA**	5.8	4.3	1.6	2.7	0.6	1.2
**LM**	1.5	2.1	0.4	0.6	1.3	1.0
**EF**	2.9	2.1	1.6	1.3	0.6	1.9
**EC**	1.5	0.8	0.4	0.3	0.6	0.5
**AB**	0.7	1.1	0.4	0.6	0.6	1.0
**KA**	0.4	0.1	0.4	0.3	0.3	0.1

**Table 7:** Composition of niosomal formulations.

Formulation Acronyms	LID (mg)	DPPC (mg)	C_14_PN(CH_3_)_3_ (mg)	C_12_PN(CH_3_)_3_ (mg)	CA (mg)
**L**	30	-	-	-	-
**L/C (9:1)**	40.5	-	-	-	4.5
**L/D (8:2)**	24	6	-	-	-
**L/C/P_12_ (7:1:2)**	31.5	-	9	-	4.5
**L/D/P_12_ (6:2:2)**	18	6	-	6	-
**L/P_14_ (9:1)**	27	-	3	-	-
**L/C/P_14_ (8:1:1)**	24	-	3	-	3
**L/C/P_14_ (7:1:2)**	21	-	6	-	3
**L/D/P_14_ (6:2:2)**	18	6	6	-	-
**L/C/D/P_14_ (6:1:2:1)**	27	9	4.5	-	4.5
**L/C/D/P_14_ (5:1:2:2)**	22.5	9	9	-	4.5
**L/C/D/P_14_ (2:1:5:2)**	9	22.5	9	-	4.5

## Data Availability

All the data are available upon reasonable request.

## Supplementary Materials

The following supporting information can be downloaded at https://www.mdpi.com/article/10.3390/molecules29122843/s1: Figure S1: ^1^H-NMR of C_12_PN(CH_3_)_3_; Figure S2: ^13^C-NMR of C_12_PN(CH_3_)_3_; Figure S3: HRMS of C_12_PN(CH_3_)_3_; Figure S4: ^1^H-NMR of C_14_PN(CH_3_)_3_; Figure S5: ^13^C-NMR of C_14_PN(CH_3_)_3_; and Figure S6: HRMS of C_14_PN(CH_3_)_3_.
