# Supplementary material for: Antimicrobial and Anesthetic Niosomal Formulations Based on Amino Acid-Derived Surfactants
Source: Molecules. 2024 Jun 14;29(12):2843. doi: 10.3390/molecules29122843 (PMC11206639; doi:10.3390/molecules29122843)
Supplement: Supplementary file 1 [file molecules-29-02843-s001.zip › molecules-3032623-supplementary.pdf]

Antimicrobial and Anesthetic Niosomal Formulations Based on Amino Acid-Derived Surfactants

Martina Romeo [1], Zakaria Hafidi [2], Rita Muzzalupo [1], Ramon Pons [2], María Teresa García [2], Elisabetta Mazzotta [1] and Lourdes Pérez [2,*]

[1] Department of Pharmacy, Health and Nutritional Sciences, University of Calabria, Via P. Bucci, 87036 Arcavacata di Rende, Italy; martina.romeo@unical.it (M.R.); rita.muzzalupo@unical.it (R.M.);
mazzotta-elisabetta@libero.it (E.M.)

[2] Department of Surfactants and Nanobiotechnology, Institute for Advanced Chemistry of Catalonia
(IQAC-CSIC), 08034 Barcelona, Spain; zhatnt@cid.csic.es (Z.H.); ramon.pons@iqac.csic.es (R.P.); teresa.garcia@iqac.csic.es (M.T.G.)

* Correspondence: lourdes.perez@iqac.csic.es

Supplementary materials:

Figure S1: 1HNMR of $C_{12}PN(CH_3)_3$

Figure S2: 13CNMR of $C_{12}PN(CH_3)_3$

Figure S3: HRMS of $C_{12}PN(CH_3)_3$

Figure S4: 1HNMR of $C_{14}PN(CH_3)_3$

Figure S5: 13CNMR of $C_{14}PN(CH_3)_3$

Figure S6: HRMS of $C_{14}PN(CH_3)_3$

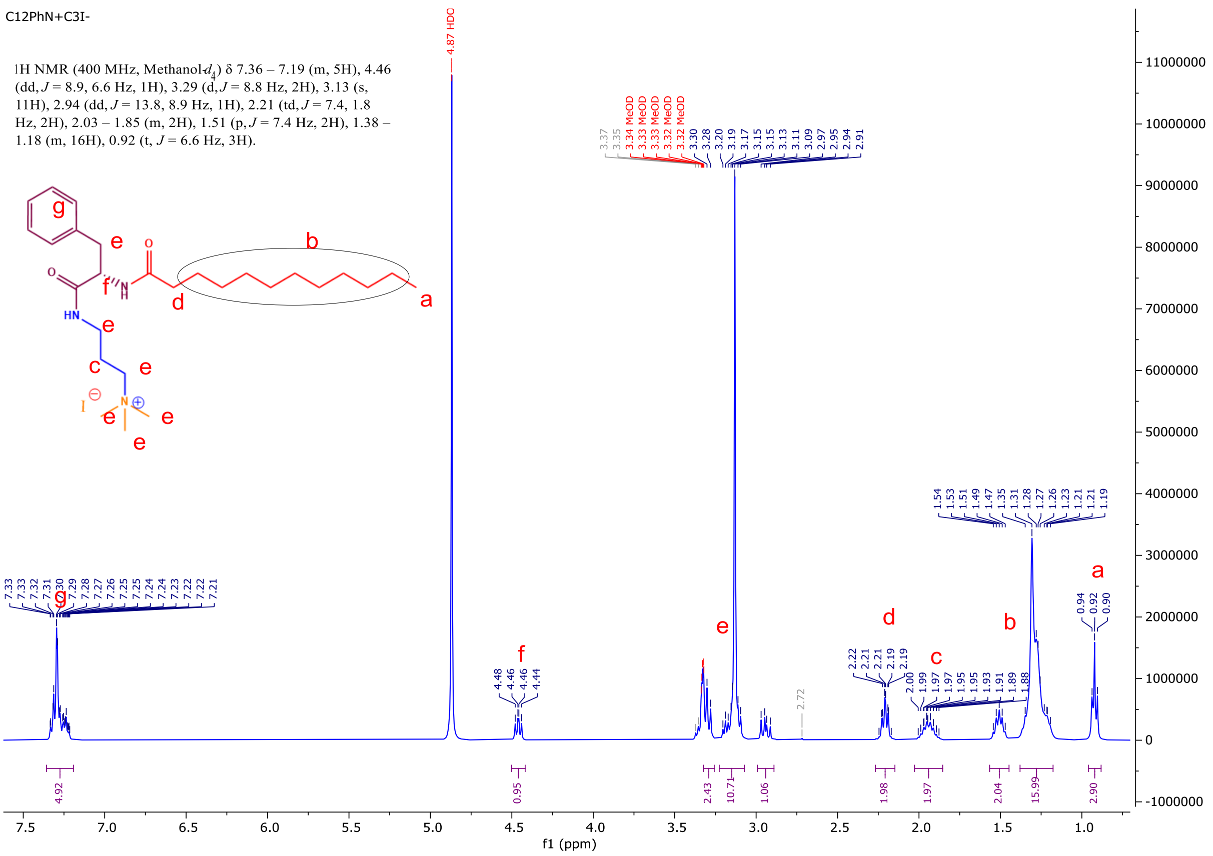

Figure S1: 1HNMR of $C_{12}PN(CH_3)_3$

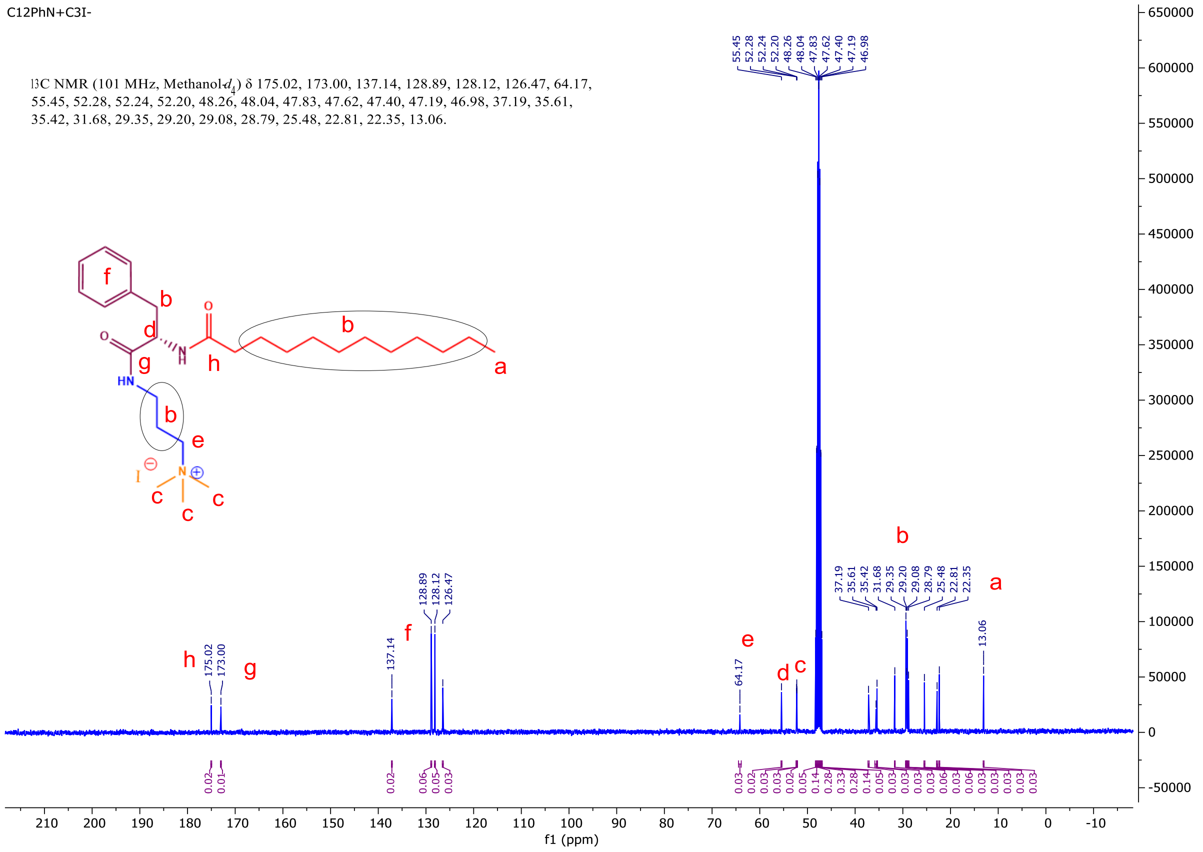

Figure S2: 13CNMR of $C_{12}PN(CH_3)_3$

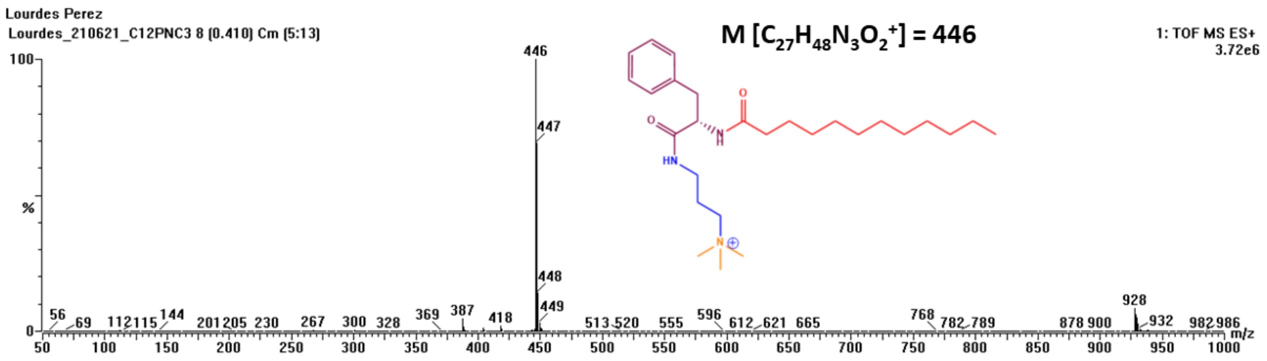

Figure S3: HRMS of $C_{12}PN(CH_3)_3$

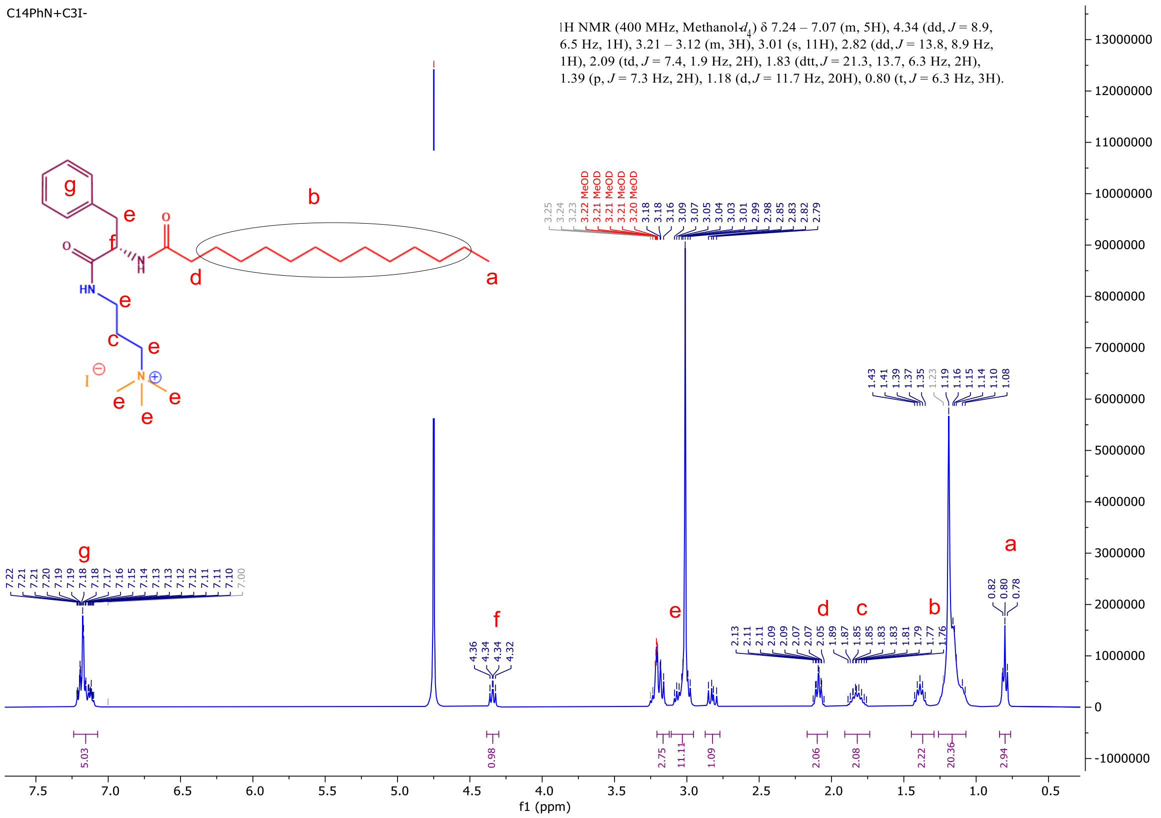

Figure S4: 1HNMR of $C_{14}PN(CH_3)_3$

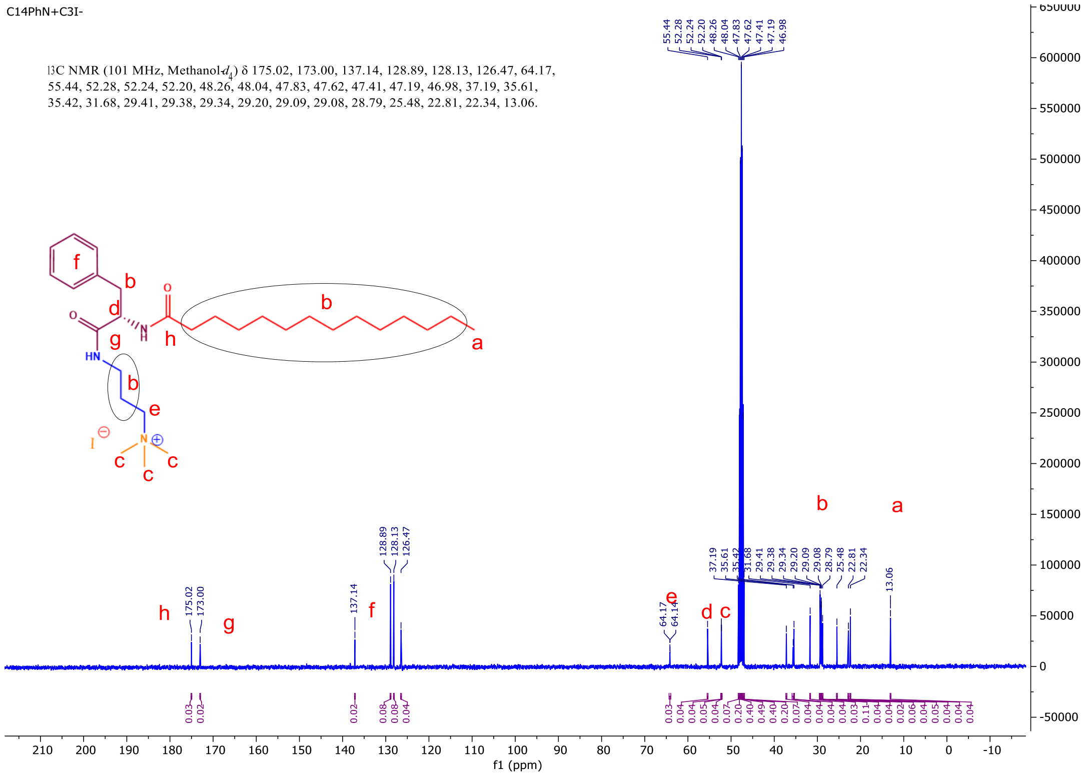

Figure S5: 13CNMR of $C_{14}PN(CH_3)_3$

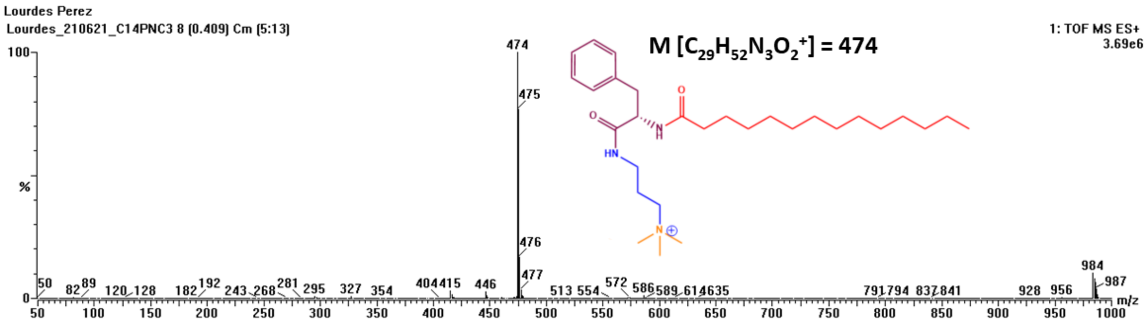

Figure S6: HRMS of C14PN(CH3)3
